# Improved jet lag recovery is associated with a weaker molecular biological clock response around the time of expected activity onset

**DOI:** 10.3389/fnbeh.2025.1535124

**Published:** 2025-01-31

**Authors:** Marie-Claire Boutrin, Melissa E. S. Richardson, Feyikemi Oriola, Samira Bolo

**Affiliations:** Department of Biological Sciences, Oakwood University, Huntsville, AL, United States

**Keywords:** jet lag recovery, circadian gene expression, master clock, day length, negative masking

## Abstract

**Introduction:**

Properly timed environmental light input to the suprachiasmatic nucleus (SCN) in the brain is crucial in maintaining the 24-hour biological rhythm (circadian rhythm). However, light exposure at the wrong time of the day-night cycle is disruptive to circadian-regulated behaviors such as the sleep-wake cycle and memory. While factors such as jet lag, variations in day length, and light at night are known disruptors to the timing of activity onset following rest, the molecular consequence of the intersection of multiple disruptions is less understood.

**Methods:**

Here, we expose mice to a jet lag paradigm under two light-dark (LD) conditions (12:12 LD and 8:16 LD) coupled with additional light exposure at night during the recovery period (known as negative masking), previously demonstrated to improve jet lag-related memory loss in mice.

**Results:**

Our results show that jet lag exposure in both LD cycles (to a greater extent in 8:16 LD) increased the fold-change of circadian gene expression in the SCN relative to the dark onset. The further addition of light during the jet lag recovery period reduced typical changes in circadian gene expression in the SCN to minimal levels under both LD cycles.

**Discussion:**

This study uncovers a novel explanation for the impact of multiple disruptive light exposures on gene expression of the molecular SCN clock in the brain.

## Introduction

Many organisms, including humans, maintain an approximately 24-h internal biological clock known as circadian rhythms that control physiological functions such as the timing of sleep, metabolic processing, regulation of hormones, and memory (Zhang et al., 2014; Astiz et al., 2019; Bolsius et al., 2021). The suprachiasmatic nucleus (SCN) of the hypothalamus of the brain is a key regulator of coordinating circadian rhythms in tissues throughout the body and is often referred to as the “master clock.” The molecular clock consists of a sequence of transcriptional events occurring during an approximate 24-h period. During that period, circadian genes such as *Per1, Per2*, and *Cry1* are temporally expressed and regulated using positive and negative feedback mechanisms to control and coordinate daily behaviors (Zhang et al., 2014). Additionally, the SCN receives input from the environment about the external 24-h day-night cycle which results in the precise fine-tuning of the SCN master clock each day.

Due to increased global travel, disruptions to the natural day-night cycle such as jet lag have become more common. Jet lag occurs because of internal circadian misalignment with the external day-night cycle and results in numerous symptoms including sleepiness, fatigue, and decreased mental sharpness (Sack et al., 2007; Sahar and Sassone-Corsi, 2009; Choy and Salbu, 2011; Savvidis and Koutsilieris, 2012; Bin et al., 2019). Seasonal changes in day length have also been shown to induce similar symptoms in humans, and since jet lag can occur at any time of the year and different latitudes on the planet, further investigation of the intersection of these two disruptions can deepen our insight on how environmental conditions can affect circadian regulated behaviors (Schaap et al., 2003; Brown et al., 2005; Rohling et al., 2006; Inagaki et al., 2007; Waterhouse et al., 2007). Studies have demonstrated that circadian gene expression in the master clock during jet lag (or different day lengths) is considerably disrupted and negatively affects cellular biology, suggesting an amplified disruptive role for jet lag and day length in this study (Lin et al., 2015; Hassan et al., 2018).

Understanding how the disruptions to the day-night environment impact circadian rhythms is crucial to designing solutions to improve synchrony between the planned arousal time and sharp cognitive and physical performance. Researchers have developed methods to reduce jet lag symptoms through interventions such as timed light exposure and pharmaceutical administration (such as melatonin), but the effectiveness is variable, and the time of year may not be accounted for in the treatment plan (Boulos et al., 1995; Oxenkrug and Requintina, 2003; Avidan and Colwell, 2010; Noyek et al., 2016). A previous study reported that jet lag recovery in mice is slower in shorter day lengths compared to longer day lengths using circadian-driven wheel-running behavior, an indicator of daily activity pattern (Richardson et al., 2020). This study also used a therapeutic approach that improved jet lag-related memory dysfunction in mice by using light exposure at night during the recovery period known as “negative masking” (Richardson et al., 2020). Understanding how day length and negative masking affect gene expression in the SCN during jet lag recovery is important for understanding how a delay in activity onset alignment with the day-night cycle during jet lag recovery may affect alertness and executive functions.

In this study, we exposed mice to a 6-h earlier shifted (advanced) jet lag paradigm under 2 days lengths: 12:12 LD (12 h light: 12 h dark or normal day length) and 8:16 LD (8 h light: 16 h dark or short day length) to study circadian gene expression in the SCN (molecular clock). We focused on the expression of seven key circadian genes including *Period* (*Per*) 1 and 2, *Cryptochrome* (*Cry*) 1 and D site-binding protein (*DBP*) at two time points relevant to circadian-driven activity onset. We also used negative masking as a tool to determine whether light exposure at night during jet lag recovery would improve any observed disruption to the gene expression pattern as previously observed with behavioral studies. Here we show that fold-changes in circadian gene expression in the SCN were amplified during jet lag in a day-length-dependent manner for specific genes. We also found that exposure to the negative masking light treatment during jet lag recovery dampened the magnitude of fold-change in gene expression associated with dark onset. Of the two tested day lengths, 8:16 LD displayed the greatest modulation in circadian gene expression during jet lag, which correlated with slow jet lag recovery and reduced memory functions, previously published in Richardson et al. (2020). These findings demonstrate the intersectional connection between day length, jet lag, and negative masking on circadian gene expression in the SCN at dark onset, providing insight on the molecular drivers of day length-related behavioral disruptions during jet lag.

## Materials and methods

### Mice

Male C57BL/6NCrl mice were used for our experiments. The age-matched male mice between 4 and 8 months old were used to minimize hormonal variation in the data (Jud et al., 2005; Datta et al., 2016). Mice were housed and treated by NIH and IACUC guidelines. Protocols used in this study were approved by the Oakwood University Animal Care and Use Committee. Three mice were used per experimental group.

### Light cycles: day lengths, jet lag, and masking paradigms

To study the effects of day length on gene expression in the molecular clock, we used 2 days length conditions: (1) 12:12 LD, which consists of 12 h of light followed by 12 h of darkness, a day length environment considered typical for circadian behavior and commonly used in experiments, and (2) 8:16 LD, which consists of 8 h of light followed by 16 h of darkness, which is not common and considered different because it represents a shorter daylight exposure than normal. Mice were all raised in 12:12 LD. Mice that were used in the 8:16 LD experiments were transitioned during adulthood for 2 weeks prior to jet lag experiments. The approach to transitioning to jet lag was aimed at providing the least disruptive method to circadian rhythms as previously published in Richardson et al. (2020). Specifically, during the transition day for the 6-h phase advance, the light period was shortened by 6 h to compensate for the initial adjustment in day length on the first day in jet lag. The negative masking light pulse was administered on the first day of jet lag during the latter part of the dark period as previously described in Richardson et al. (2020).

### Tissue collection

To capture changes in circadian gene expression relative to dark onset, which is relevant to the start of circadian-driven activity (expected at ZT12), we used two well-established time points where the molecular clock is known to be sensitive to environmental perturbations. These two time points are (1) Z*eitgeber 10* (ZT10), which is described by circadian biologists as 2 h before darkness begins, and (2) Z*eitgeber 14* (ZT14), which is described by circadian biologists as 2 h after darkness begins (Karatsoreos and Silver, 2017). Mice were euthanized by cervical dislocation at the set time points and the brains immediately dissected under a dissecting microscope to retrieve SCN tissue samples. For the non-jet lag (NJL) control experiments, mice with stable circadian rhythms (at least 14 days in the 12:12 LD or 8:16 LD cycle without perturbations) were sacrificed at ZT10 or ZT14. To model the jet lag (JL) paradigm in our study, we used a 6-h shift earlier (advancement) in the LD cycle starting with 12:12 LD. Since our previous study demonstrated that jet lag recovery was quickened following the addition of a light pulse of 4 h (referred to herein as jet lag with negative masking or JL + MSK), we replicated the same paradigm to study the impact of environmental changes on circadian gene expression patterns in the SCN relative to the dark onset. For JL and JL + MSK experiments, mice were sacrificed after the second day began (completion of the first full cycle and start of the next) at ZT10 or ZT14. SCN tissue samples were freeze-dried using liquid nitrogen and stored at −80°C until they were processed for Real-Time PCR analysis.

### Real-Time PCR analysis

Real-Time PCR analysis was used to study gene expression levels. Total RNA samples were obtained from dissected SCN mouse tissue using the SV Total RNA Isolation System kit as per manufacturer’s instructions (Promega, WI). cDNA was synthesized using a GoScript^TM^ Reverse Transcription System kit (Promega, WI). cDNA samples were processed using a QuantiTect SYBR Green PCR kit (Qiagen, CA) and QuantStudio 5 instrument (Thermo Fisher Sientific, MA). The genes studied belong to the three important components of the molecular clock: the *Per-Cry* loop, the *CLOCK-Bmal1* loop, and the *DBP* and *Reverb-α* genes. The list of primers for the tested genes is available in the Supplementary material. The 2^−ΔΔCT^ (relative) method was used for data analysis and referenced to the *18 s* gene, as described in Livak and Schmittgen (2001). A 1.5-fold change in gene expression was considered a modulation in gene expression. We determined fold changes in gene expression between ZT10 (reference point) and ZT14 under NJL, JL, and JL + MSK conditions under the 12:12 LD and 8:16 LD day lengths for each circadian gene: *Per1-2, Cry1, Bmal1, CLOCK, Reverb-*α, and *DBP*. The evaluation of gene expression at ZT10 and ZT14 provides measurements of transcriptional activity during the light and darkness phases, 2 h before and after activity is expected to begin, respectively.

### Data and statistical analysis

Three mice samples per experimental group were used for our analyses. The T-test was used for the comparison of two experimental groups, and the One-Way ANOVA test was used for analyses comparing more than two experimental groups, followed by a Tukey post-hoc test for additional information on differences between groups. Statistical analysis was done using the GraphPad Prism version 10.0.0, GraphPad Software (MA, www.graphpad.com). Statistical significance was set at *p* < 0.050.

## Results

### The gene expression profile in the SCN was comparable to previous studies and partially affected by day length

**Figure 1:**
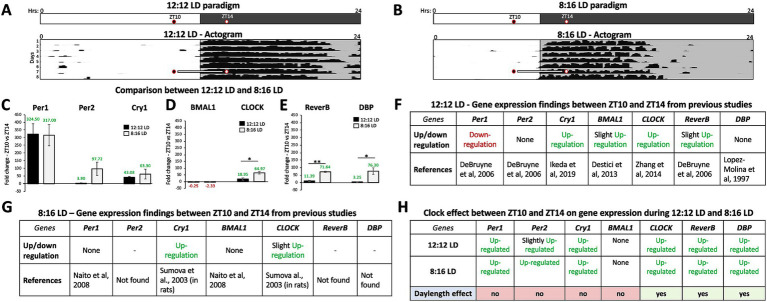
Gene expression in the SCN varies with day length in a circadian-dependent manner. **(A)** Schematic of the 12:12 LD paradigm with the times of tissue extraction is indicated by two circles for ZT10 (2 h before dark onset) and ZT14 (2 h after dark onset). A representative actogram (Adapted from Simmonds, 2015) depicts the circadian alignment of activity with dark onset during 12:12 LD and the times (ZT 10 and 14) of SCN tissue extraction. **(B)** Schematic of the 8:16 LD paradigm with ZT10 (2 h before dark onset) and ZT14 (2 h after dark onset). A representative actogram (Adapted from Simmonds, 2015) depicts the circadian alignment of activity with dark onset during 8:16 LD and the times (ZT 10 and 14) of SCN tissue extraction. Comparison of average fold-change in gene expression between ZT10 and ZT14 during 12:12 LD and 8:16 LD before jet lag (control conditions) for **(C)** *Per1*, *Per2*, and *Cry1.* **(D)** *Bmal1* and *CLOCK.* **(E)** *Reverb-α* and *DBP.* **(F)** Comparison of gene expression patterns between the current study and previously published data during 12:12 LD between ZT10 and ZT14. **(G**) Comparison of gene expression patterns between the current study and previously published data during 8:16 LD between ZT10 and ZT14. **(H)** Circadian variations (ZT10 vs. ZT14) in gene expression and effect of day length (12:12 LD and 8:16 LD). n = 3 for each experimental group. Error bars are ± Standard Deviation. **p < 0.01, ***p < 0.001.

Fold changes in circadian gene expression between ZT10 and ZT14 were compared during 12:12 LD and 8:16 LD before jet lag (control conditions) and are as follows (Figure 1): (C) *Per1* (12:12LD: 324.50 ± 67.15; 8:16LD: 317.00 ± 68.93; t_4_ = 0.1349, *p* = 0.8992), *Per2* (12:12LD: −3.90 ± 2.68; 8:16LD: 97.72 ± 43.66; t_2.015_ = 3.714, *p* = 0.0647), and *Cry1* (12:12LD: 43.07 ± 4.21; 8:16LD: 63.29 ± 31.93; t_2.070_ = 1.088, *p* = 0.3871). (D) *Bmal1* (12:12LD: −0.25 ± 2.39; 8:16LD: −2.33 ± 1.27; t_4_ = 1.327, *p* = 0.2552) and *CLOCK* (12:12LD: 18.95 ± 8.65; 8:16LD: 64.97 ± 21.62; t_4_ = 3.423, *p* = 0.0267) and (E) *Reverb-α* (12:12LD: −11.39 ± 4.17; 8:16LD: 71.64 ± 21.39; t_4_ = 4.787, *p* = 0.0087) and *DBP* (12:12LD: 3.25 ± 1.93; 8:16LD: 76.30 ± 22.18; t_2.030_ = 5.683, *p* = 0.0286). Except for *BMAL1*, the fold-change in gene expression for the *Per-Cry* loop, the *CLOCK*, and *DBP* and *Reverb-α* were up-regulated at ZT14 relative to ZT10 during 12:12 LD and 8:16 LD conditions (Figures 1C–E). When considering the means and standard variations for our results, we find them to be comparable to previous findings for both day lengths, except for *Per1* in 8:16 LD, which showed no significant difference in gene expression fold changes between ZT10 and ZT14 in a previous study (we have an up-regulation; Figure 1D; Naito et al., 2008).

Fold changes in gene expression recorded under 12:12 LD and 8:16 LD for the *Per-Cry* loop, *Bmal1-CLOCK* loop, and *Reverb-α* and *DBP* group were compared using unpaired T-test analysis and were found to be statistically and significantly higher under 8:16 LD for *CLOCK* (46.02 ± 13.44; t_4_ = 3.423, *p* = 0.0267), *Reverb-α* (60.24 ± 12.58; t_4_ = 4.787, *p* = 0.0087), and *DBP* (73.05 ± 12.86; t_2.03_ = 5.683, *p* = 0.0286) compared to those under 12:12 LD. Our findings reveal that the patterns of regulation for the *CLOCK-Bmal1* loop and *Reverb-α* and *DBP* vary with day length (more up-regulated in 8:16 LD; Figure 1H).

### Jet lag and negative masking exposures impact circadian gene expression by both up-regulation and down-regulation in the SCN under 12:12 LD around dark onset

**Figure 2:**
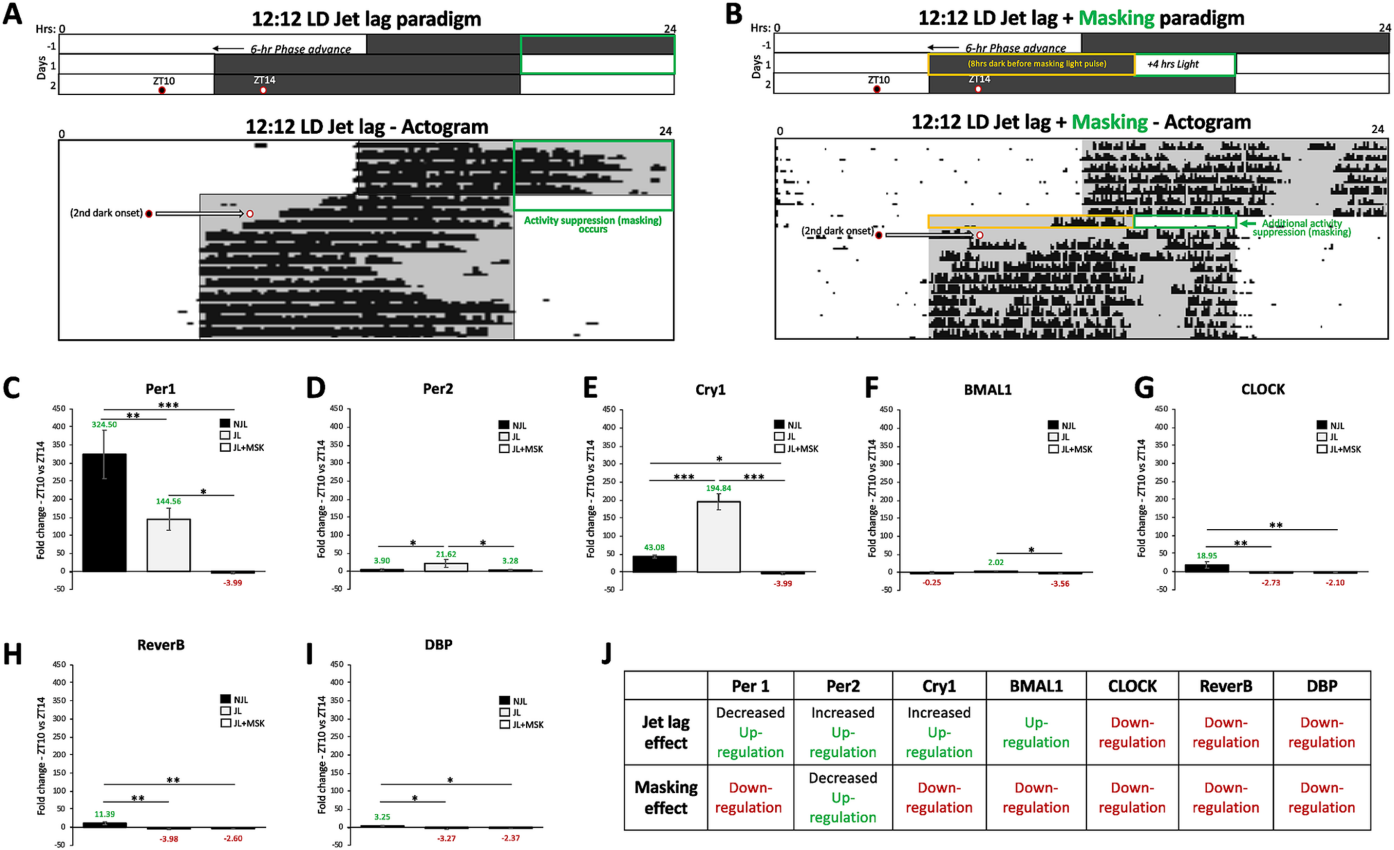
Jet lag and negative masking exposures impact circadian gene expression in the SCN under 12:12 LD around dark onset. **(A)** Schematic of the 12:12 LD jet lag paradigm with the 6-h phase advancement (earlier shifted) day-night cycle. A representative actogram (Adapted from Richardson et al., 2020) depicts the circadian alignment of activity with dark onset during 12:12 LD jet lag. Two circles indicate the times of tissue extraction at ZT10 and ZT14 before and after the second dark onset of the jet lag paradigm. **(B)** Schematic of the 12:12 LD jet lag paradigm with the 4-h of light added to the end of the first night of jet lag (the masking paradigm – green box). A representative actogram (Adapted from Richardson et al., 2020) depicts the circadian alignment of activity with dark onset during 12:12 LD jet lag + masking (4 h of light). Two circles indicate the times of tissue extraction at ZT10 and ZT14 before and after the second dark onset of the jet lag + masking paradigm. The orange box indicates the 8-h dark period on the first day of jet lag preceding the masking light exposure. Comparison of average fold-change in gene expression between ZT10 and ZT14 during 12:12 LD before jet lag (NJL—control conditions), during jet lag (JL), and jet lag + masking (JL + MSK) for **(C)** *Per1*; **(D)** *Per2*; **(E)** *Cry1*; **(F)** *Bmal1*; **(G)** *CLOCK*; **(H)** *Reverb-α*; **(I)** *DBP.* **(J)** Circadian variations (ZT10 vs. ZT14) in gene expression and effect of jet lag and masking during 12:12 LD. n = 3 for each experimental group. Error bars are ± Standard Deviation. *p < 0.05, **p < 0.01, ***p < 0.001.

We compared the average fold change patterns in gene expression between ZT10 and ZT14 under NJL (control conditions), JL, and JL + MSK conditions under the 12:12 LD day length for each circadian gene: *Per1-2, Cry1, Bmal1, CLOCK, Reverb-α*, and *DBP.* The results are as follows (Figure 2): (C) *Per1* (NJL: 324.50 ± 67.15; JL: 144.56 ± 30.52; JL + MSK: −3.99 ± 2.30; *F*(2,6) = 44.71, *p* < 0.001); (D) *Per2* (NJL: 3.90 ± 2.68; JL: 21.61 ± 11.20; JL + MSK: 3.27 ± 1.30; *F*(2,6) = 7.250, *p* = 0.0251; (E) *Cry1* (NJL: −43.07 ± 4.21; JL: 194.84 ± 22.48; JL + MSK: −3.98 ± 3.19, *F*(2,6) = 182.2, *p* < 0.001); (F) *BMAL1* (NJL: −0.25 ± 2.39; JL: 2.02 ± 0.89; JL + MSK: −3.55 ± 0.97; *F*(2,6) = 9.481, *p* = 0.0139); (G) *CLOCK* (NJL: 18.94 ± 8.65; JL: −2.73 ± 1.53; JL + MSK: −2.09 ± 1.23; *F*(2,6) = 17.39, *p* = 0.0032); (H) *Reverb-α* (NJL: 11.39 ± 4.17; JL: −3.98 ± 1.91; JL + MSK: −2.60 ± 1.94; *F*(2,6) = 26.21, *p* = 0.0011) and (I) *DBP* (NJL: 3.24 ± 1.92; JL: −3.26 ± 2.70; JL + MSK: −2.37 ± 0.75, *F*(2,6) = 9.671, *p* = 0.0133).

The results reveal that under JL conditions, the expression of *Per1*, *CLOCK, Reverb-α* and *DBP* was down-regulated, whereas the expression of *Per2*, *Cry1*, *Bmal1* was up-regulated (Figures 2C–I). The addition of the negative masking light exposure on the first day of jet lag resulted in a down-regulation in the expression of all tested genes compared to JL levels, except for *CLOCK*, *Reverb-*α and *DBP* that showed no statistically significant difference in expression levels between JL and JL + MSK (Figure 2C–I, summarized in 2 J). One-Way ANOVA test showed that the fold changes in circadian gene expression obtained for NJL, JL and JL + MSK exposures were statistically significantly different (*p* < 0.05) for most of the genes, at the exception of *Per2*, *Bmal1, CLOCK*, *Reverb-*α and *DBP* for a few conditions (Figures 2C–I). A Tukey post hoc test revealed that the fold change in gene expression was not statistically significantly different between NJL and JL + MSK for *Per2* (0.63 ± 5.47, *p* = 0.9928) and *Bmal1* (3.31 ± 1.29, *p* = 0.0939), and between NJL and JL for *Bmal1* (−2.27 ± 1.29, *p* = 0.2589). The Tuckey post-hoc also showed that expression levels did not statistically significantly differ during the JL and JL + MSK for *CLOCK*, *Reverb-*α and *DBP* (−0.63 ± 4.18, *p* = 0.9875; −2.60 ± 2.35, *p* = 0.8319; −0.89 ± 1.60, *p* = 0.8471, respectively). These findings demonstrate that during 12:12 LD, both JL and JL + MSK conditions impact circadian gene expression in the SCN by both down- or up-regulation around dark onset (Figure 2J).

### Jet lag and negative masking exposures affect circadian gene expression by both up-regulation and down-regulation in the SCN under 8:16 LD around dark onset

**Figure 3:**
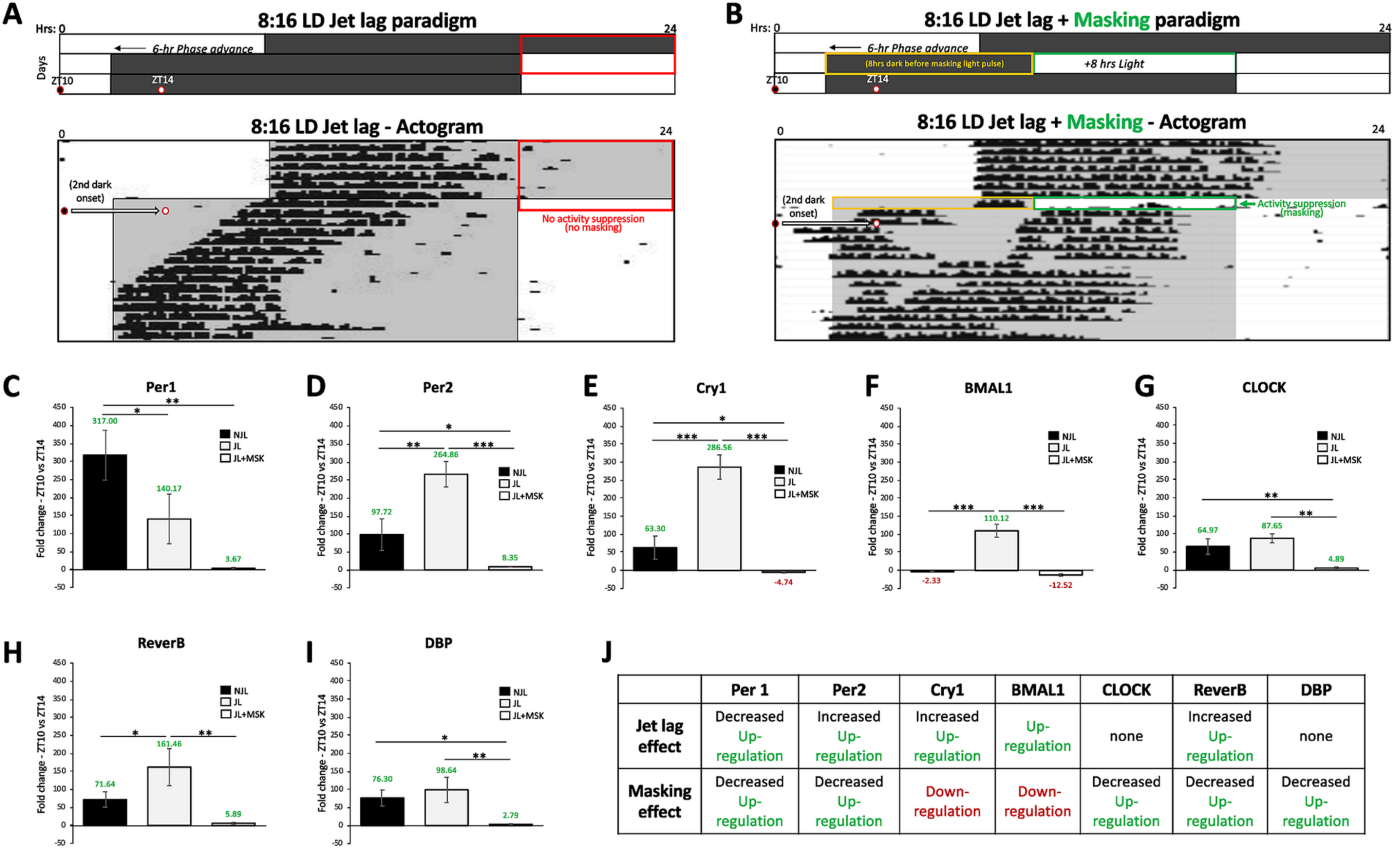
Jet lag and negative masking exposures impact circadian gene expression in the SCN under 8:16 LD around dark onset. **(A)** Schematic of the 8:16 LD jet lag paradigm with the 6-h phase advancement (earlier shifted) day-night cycle. A representative actogram (Adapted from Richardson et al., 2020) depicts the circadian alignment of activity with dark onset during 8:16 LD Jet lag. Two circles indicate the times of tissue extraction at ZT10 and ZT14 before and after the second dark onset of the jet lag paradigm. **(B)** Schematic of the 8:16 LD jet lag paradigm with the 8-h of light added to the end of the first night of jet lag (the masking paradigm – green box). A representative actogram (Adapted from Richardson et al., 2020) depicts the circadian alignment of activity with dark onset during 8:16 LD jet lag + masking (8 h of light). Two circles indicate the times of tissue extraction at ZT10 and ZT14 before and after the second dark onset of the jet lag + masking paradigm. The orange box indicates the 8-h dark period on the first day of jet lag preceding the masking light exposure. Comparison of average fold-change in gene expression between ZT10 and ZT14 during 8:16 LD before jet lag (NJL—control conditions), during jet lag (JL), and jet lag + masking (JL + MSK) for **(C)** *Per1*; **(D)** *Per2;* **(E)** *Cry1;* **(F)** *BMAL1*; **(G)** *CLOCK*; **(H)** *Reverb-α* and **(I)** *DBP*. **(J)** Circadian variations (ZT10 vs. ZT14) in gene expression and effect of jet lag and masking during 8:16 LD. n = 3 for each experimental group. Error bars are ± Standard Deviation. *p < 0.05, **p < 0.01, ***p < 0.001.

Similar to our approach used for 12:12 LD in Figure 2, we studied the impact of jet lag and negative masking light exposure paradigm under 8:16 LD conditions at ZT10 and ZT14 (Figures 3A,B). Under JL conditions, all tested genes exhibited up-regulation, many of which were exaggerated in comparison to the NJL conditions (Figures 3C–I). The addition of the negative masking light exposure on the first day of jet lag resulted in a down-regulation of *Cry1* and *Bmal1* expression and a decreased up-regulation of the other tested genes, compared to JL (Figures 3C–I). The One-Way ANOVA test showed that fold changes in circadian gene expression obtained for NJL, JL, and JL + MSK exposures were statistically significantly different for all genes, with a few exceptions (Figures 3C–I). The results for the One-Way ANOVA test comparing average fold-change in gene expression between ZT10 and ZT14 during 8:16 LD before JL (NJL—control conditions), during JL, and JL + MSK for Figure 3C–I are as follows: (C) *Per1* (NJL: 317.00 ± 68.93; JL: 140.17 ± 69.11 JL + MSK: 3.67 ± 2.33; *F*(2,6) = 23.30, *p = 0*.0015); (D) *Per2* (NJL: 97.72 ± 43.66; JL: 264.86 ± 35.37; JL + MSK: 8.35 ± 0.82; *F*(2,6) = 48.31, *p* < 0.001); (E) *Cry1* (NJL: 63.30 ± 31.93; JL: 286.56 ± 33.92; JL + MSK: −4.74 ± 2.68), *F*(2,6) = 96.01, *p* < 0.001); (F) *BMAL1* (NJL: −2.33 ± 1.27; JL: 110.12 ± 18.16; JL + MSK: −12.52 ± 3.41; *F*(2,6) = 121.6, *p* < 0.001); (G) *CLOCK* (NJL: 64.97 ± 21.62; JL: 87.65 ± 12.53; JL + MSK: 4.89 ± 3.03; *F*(2,6) = 25.98, *p = 0*.0011); (H) *Reverb-α* (NJL: 71.64 ± 21.39; JL: 161.46 ± 51.30; JL + MSK: 5.88 ± 2.60; *F*(2,6) = 17.73, *p* = 0.0030); and (I) *DBP* (NJL: 76.30 ± 22.18; JL: 98.64 ± 35.12; JL + MSK: 2.79 ± 1.89; *F*(2,6) = 13.09, *p* = 0.0065). These results show that fold changes in circadian gene expression were generally statistically significantly higher under JL compared to NJL, except for *CLOCK* and *DBP* (−22.67 ± 11.86, *p* = 0.2158; −22.34 ± 19.60, *p* = 0.5270, respectively). The Tukey post-hoc test also reported all fold changes in gene expression to be statistically significantly lower under the JL + MSK condition compared to JL, except for *Per1* (176.8 ± 46.02, *p* = 0.0567; Figures 3C–I). Additionally, the Tukey post-hoc test revealed *Bmal1* and *Reverb-α* expression under NJL and JL + MSK was not statistically significantly different (10.19 ± 8.73, *p* = 0.5121; 65.75 ± 26.23, *p* = 0.1012, respectively). These findings demonstrate that during 8:16 LD, both JL and JL + MSK conditions impact circadian gene expression in the SCN by both down- or up-regulation around dark onset (Figure 3J).

### Day length influences SCN circadian gene response to masking in jetlag recovery

**Figure 4:**
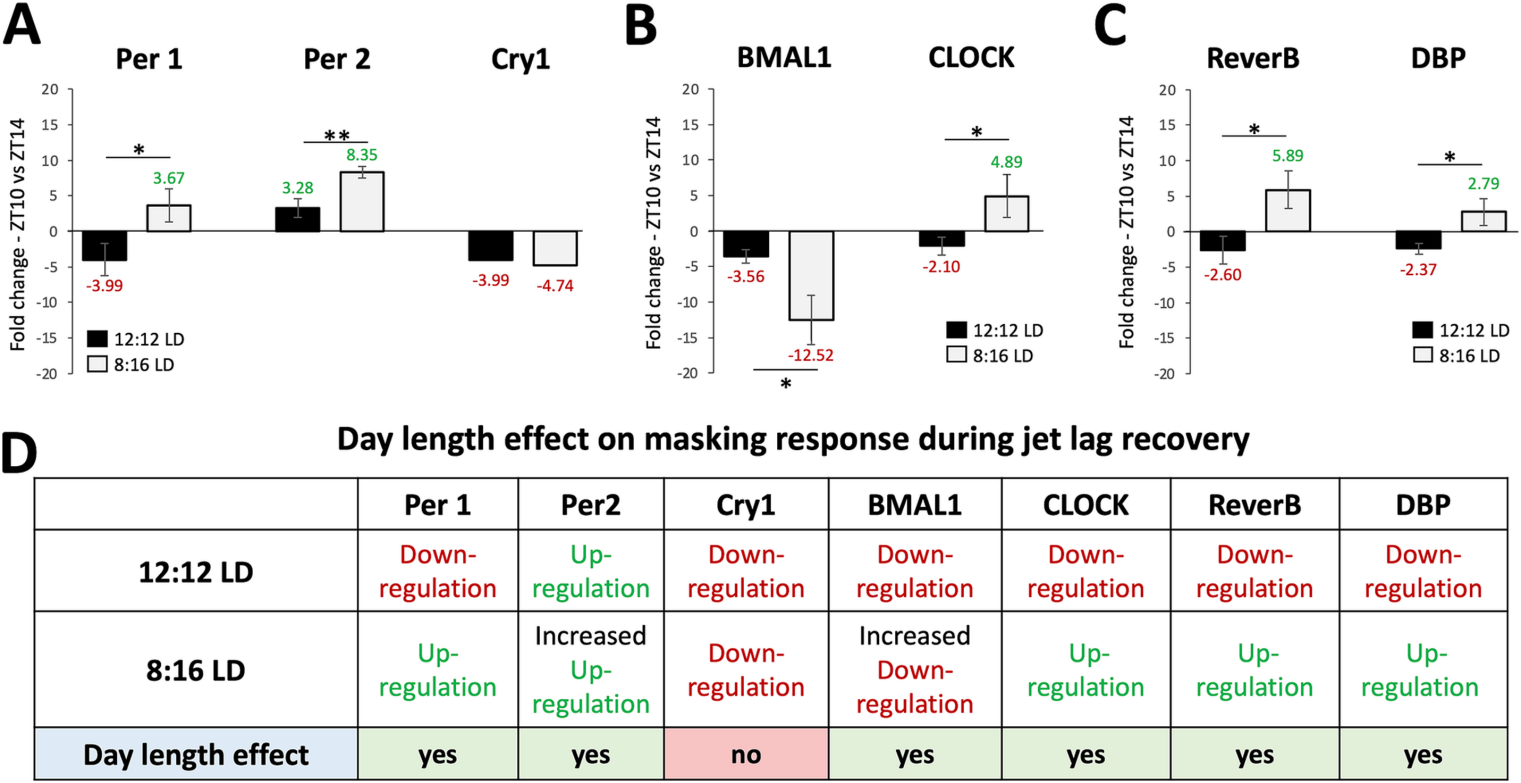
The masking paradigm improved the gene expression outcomes during jet lag with unique patterns in each day length. Comparison of average fold-change in gene expression between ZT10 and ZT14 during 12:12 LD and 8:16 LD during jet lag + masking (JL + MSK) for **(A)** *Per1*, *Per2*, and *Cry1*; **(B)** *BMAL1* and *CLOCK;* **(C)** *Reverb-α* and *DBP*. **(D)** Circadian variations (ZT10 vs. ZT14) in gene expression and effect of daylength (12:12 LD and 8:16 LD) during JL + MSK. n = 3 for each experimental group. Error bars are ± Standard Deviation. **p < 0.01, ***p < 0.001.

**Figure 5:**
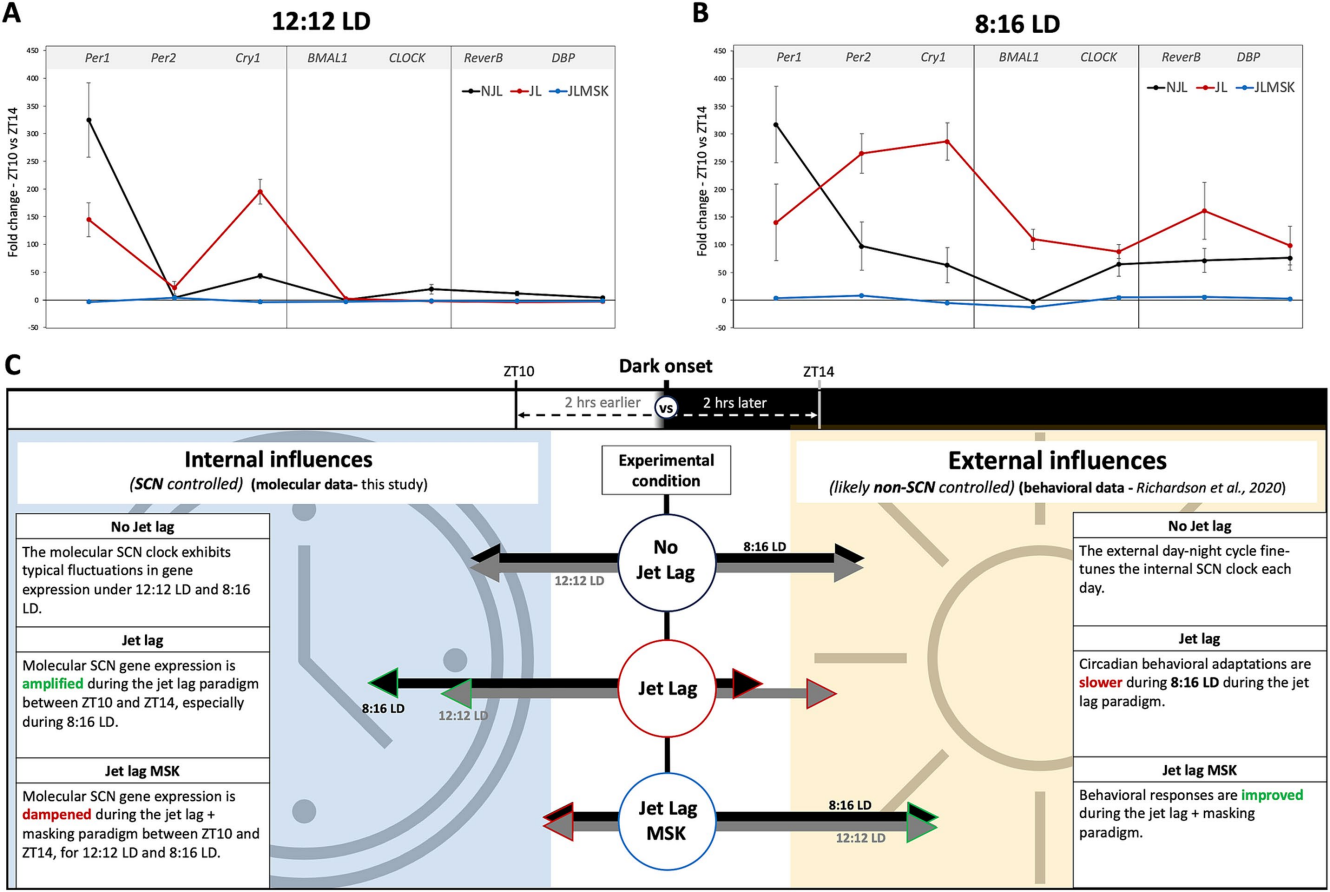
Summary of the fold-change in gene expression near dark onset under experimental conditions and a comparison of hypothetical contributions to behavioral observations during jet lag recovery. **(A)** Fold-changes in gene expression of circadian gene under 12:12 LD replotted from Figures 1–3 for overall comparison between environmental conditions. **(B)** Fold-changes in gene expression of circadian gene under 8:16 LD replotted from Figures 1–3 for overall comparison between environmental conditions. **(C)** Graphic illustrating the hypothetical contribution of circadian genes to robust circadian-driven behavior under NJL, JL, and JL + MSK, with the assumption that low SCN circadian gene expression indicates a non-SCN source controlling observed behaviors.

To determine whether the masking effects on jet lag recovery varied with day-length, we compared the JL + MSK results for each gene between 12:12 LD and 8:16 LD. The results for the unpaired T-test analysis comparing average fold-change in gene expression between ZT10 and ZT14 during 12:12 LD and 8:16 LD during JL + MSK for Figure 4 are: (A) *Per1* (12:12LD: −3.99 ± 2.30; 8:16LD: 3.67 ± 2.33; t_4_ = 4.052, *p* = 0.0155), *Per2* (12:12LD: 3.28 ± 1.31; 8:16 LD: 8.35 ± 0.82; t_4_ = 5.688, *p = 0*.0047), and *Cry1* (12:12LD: −3.99 ± 3.20; 8:16LD: −4.74 ± 2.68; t_4_ = 0.3149, *p* = 0.7686); (B) *BMAL1* (12:12LD: −3.56 ± 0.97; 8:16LD: −12.52 ± 3.41; t_4_ = 4.383, *p = 0*.0118 and *CLOCK* (12:12LD: −2.10 ± 1.23; 8:16LD: 4.89 ± 3.03; t_4_ = 3.695, *p* = 0.0209; and (C) *Reverb-α* (12:12LD: −2.60 ± 1.94; 8:16LD: 5.89 ± 2.60; t_4_ = 4.530, *p* = 0.0106) and *DBP* (12:12LD: −2.37 ± 0.76; 8:16LD: 2.79 ± 1.89; t_4_ = 4.391, *p* = 0.0118; *F*(3,8) = 89.177, *p* < 0.001). These results show that, under 12:12 LD, JL + MSK statistically significantly decreased the expression of all the tested circadian genes compared to 8:16 LD, except for *Bmal1,* which showed a significant decrease in expression under 8:16 LD (the difference in expression for *Cry1* was not statistically significant; Figures 4A–D). Therefore, our results revealed that day length changed the effect of masking on circadian gene expression under JL conditions. A comparison of all three conditions (NJL, JL, JL + MSK) under 12:12 LD and 8:16 LD demonstrates the large dampening effect of the JL + MSK treatment (Figures 5A,B). Our resulting hypothesis is that JL conditions induce more molecular activity in the circadian genes while the combination JL + MSK condition relies less on the molecular clock to drive behavioral adaptations previously observed using locomotor activity (Figure 5C and Richardson et al., 2020).

## Discussion

Our study demonstrates that clock gene expression changes either slightly or dramatically depending on the timing and duration of disruptive light exposure. Specifically, in 8:16 LD, we found a larger change in the amplitude of circadian gene expression level following JL compared to 12:12 LD. Interestingly, when mice were given an additional light pulse (negative masking) with the hypothesis that gene expression levels would return to the pre-JL gene expression amplitude, we instead found a dampening of the gene expression amplitude. This indicates a reduced molecular clock response during the JL + MSK paradigm. Since activity alignment with dark onset reflects normal behavior in nocturnal animals such as mice, studying the fluctuation is gene expression before and after dark onset allows us to determine how the SCN regulates adaptation to changing environments. Therefore, to determine the gene expression amplitude, we compared the fold changes in circadian gene expression between ZT10 and ZT14, which have been previously validated as two time points with high enough gene expression to be measured and prevent false negative results (Karatsoreos and Silver, 2017). Our results confirmed the patterns of circadian gene expression between ZT10 and ZT14 in 12:12 LD and 8:16 LD comparable to previous studies (Lopez-Molina et al., 1997; Sumová et al., 2003; DeBruyne et al., 2006; Naito et al., 2008; Destici et al., 2013; Zhang et al., 2014; Ikeda et al., 2019). Thus, selecting ZT10 and ZT14 as our circadian comparison time points is both relevant and robust to determine the response of the molecular clock to environmental light perturbations around dark onset when circadian-driven activity alignment normally occurs.

This study investigated the effects of 12:12 LD and 8:16 LD day-night cycles on the molecular circadian clock because it was previously demonstrated that jet lag recovery of locomotor rhythms was slower during 8:16 LD compared to 12:12 LD (Richardson et al., 2020). Thus, we hypothesized that the molecular circadian clock would exhibit a more significant change in gene expression to reflect the behavioral observations from the Richardson et al. (2020) study. Our results show that there is a day length effect for *CLOCK*, *Reverb-α,* and *DBP* as 8:16 LD increases the level of their expression, compared to normal day length (12:12 LD). While other studies such as the one recently published by Cox et al. (2024) show incongruent gene expression patterns compared to our data, the Cox study was performed under constant darkness, following exposure to either 16:8 LD (long day) or 8:16 LD (short day) conditions. This strengthens our conclusions that studying circadian genes under different day lengths, relative to dark onset, and with additional perturbations, is a valid model to understand how changes in the light–dark environment impact circadian gene expression. Since the *CLOCK*, *Reverb-α* and *DBP* portion of the molecular clock is more active in 8:16 LD environment, potential strategies to recover from jet lag between 12:12LD and 8:16 LD could target these genes.

Our study demonstrated how day length, and additional light exposure (negative masking) during jet lag impact SCN clock gene expression at the day-to-night transition portion of the 24-h cycle. We observed two intriguing phenomena: (1) Jet lag maintains or amplifies the pattern of pre-jet lag gene expression in a day-length dependent manner (except for *Per1*) and (2) Additional light pulse during jet lag results in the dampening of the expression pattern for specific circadian genes or a reversal of gene expression pattern from up-regulation to down-regulation in a day-length dependent manner. While we do not observe a complete loss of gene expression during the JL + MSK condition, we characterize the phenotype as a “dampening effect” because there is a minimal fluctuation in gene expression fold changes (Mean: −2.19; Range: −3.99–3.28 for 12:12 LD and Mean: 1.18; Range: −12.52–8.35 for 8:16 LD). There is an even more apparent dampening effect when the difference in gene expression fold change is compared between JL and JL + MSK (Mean = 92; Range: 5.56–198.83 for 12:12 LD and Mean = 163; Range: 82.76–291.3 for 8:16 LD). We hypothesize that during 8:16 LD, the more molecularly expressive SCN presents a greater challenge to adapt to a new day-night onset time during jet lag, contributing to the previously reported slower jet lag recovery and decreased memory aptitudes (Richardson et al., 2020). Additionally, we hypothesize that a dampening in the molecular response occurs to allow faster environmental adaptation likely using a non-SCN clock mechanism, which is congruent with previous findings that the SCN is necessary for the expression of circadian rhythms, but not for masking behavior to light (Gall and Shuboni-Mulligan, 2022), which may be responsible for the quicker recovery phenotypes previously observed (Richardson et al., 2020). We also propose this hypothesis based on previously published data where the SCN circadian clock is molecularly (*Bmal1* and *Per2/Cry1* double mutants) or behaviorally (gradual exposure to the fragmented day-night cycle, FDN-G) disrupted and rapid adaptation to changes in the light–dark environment is observed (Abraham et al., 2006; Bittman, 2021; Li and Androulakis, 2021; Richardson et al., 2023). Thus, our study indicates that a dampened gene expression response surrounding the crucial period of activity onset is associated with rapid adaptation to the jet lag paradigm.

### Limitations of the study

Although the study was successful at revealing significant findings, it has some limitations. To reach a balanced approach between reliability of results and reasonable use of mice, the study involved a small sample size because of the notable number of experimental conditions to be studied. Although the sample size was small, statistically significant results were obtained. Also, the following was done to promote the accuracy of the study’s results: (1) standardized and validated methods were used during sample analysis, data collection and analysis; (2) data quality was reviewed before moving on to the next stage of analysis and experiments were re-started from scratch with new tissue samples if needed.

## Conclusion and future directions

Our study shows that disruptions to the day-night cycle can either amplify or dampen changes in circadian gene expression in the SCN relative to the time of activity onset in mice. This finding significantly improves the approach needed to design tailored therapeutic solutions based on the connection between the external light–dark environment and the circadian gene response. We used two time points (ZT10 and ZT14) that straddle the transition point from light to dark, which is comparable between different day lengths and is relevant to capture changes necessary for activity onset. We found that not all tested circadian genes showed changes in expression between ZT10 and ZT14, which is expected due to the staggered responses of the different loops of the molecular clock across the day (Cox and Takahashi, 2019; Ikeda et al., 2019; Xie et al., 2019). We are aware that circadian studies typically assess gene expression at multiple time points across the 24-h day, which we believe is the next step in further exploring the circadian rhythms associated with jet lag recovery and therapeutic strategies under different day lengths. How the brain responds to numerous and potentially conflicting environmental inputs is crucial to predicting molecular, physiological, and behavioral outcomes for real-world applications.

## Data Availability

The datasets presented in this study can be found in online repositories. The names of the repository/repositories and accession number(s) can be found in the article/Supplementary material.
