# Supplementary material for: Improved jet lag recovery is associated with a weaker molecular biological clock response around the time of expected activity onset
Source: Front Behav Neurosci. 2025 Jan 31;19:1535124. doi: 10.3389/fnbeh.2025.1535124 (PMC11825751; doi:10.3389/fnbeh.2025.1535124)
Supplement: Supplementary file 1 [file Table_1.DOCX]

Supplementary Table 1. List of primers for the studied circadian genes

​

| **​Gene ID** | **​Forward Primer Sequence** | **​Reverse Primer Sequence** |
| --- | --- | --- |
| *​Per1* | ​CC TCC TAC ACT GCC TCT T | ​CA CGC TCT CTG CCT TAT T |
| *​Per2* | ​TTC CAG GCT GTG GAT GAA AG | ​GCG GAA TCG AAT GGG AGA ATA |
| *​Cry1* | ​GCT GGG AAG AAG GGA TGA A | ​GAA GCC TCT TAG GAC AGG TAA A |
| *​CLOCK* | ​CT CTG GAC TGC TTC TGT ATG | ​CT CTC ACA GTC TCG TCT CTA A |
| *​Bmal1* | ​AA GAC TGG ACT TCC GGT TAA A | ​TA GCC TGT GCT GTG GAT TG |
| *​Reverb-α* | ​ GT CGC TGA CAC TAC ACA GG | ​ CA GGT GGT GAA GGT ATC TCC |
| *​DBP* | ​ GCT TGA CAT CTA GGG ACA CAC | ​ TG GAC TTT CCT TGC CTT CTT |
| *​18s* | ​GT CCC TTG CCC TTT GTA CAC A | ​AT CCG AGG GCC TCA CTA AAC |

​​
